# From Colloids to Hydrogels: Concentration-Dependent Cytocompatibility of Carboxylated Cellulose Nanocrystals Prepared via Deep Eutectic Solvents

**DOI:** 10.1021/acs.langmuir.6c02021

**Published:** 2026-06-09

**Authors:** Raúl Ortega-Córdova, Griselda Blanco-Gutiérrez, Priscila Quiñonez-Angulo, Francisco J. Flores-Ruiz, Kaori Sánchez-Carrillo, J. Félix Armando Soltero-Martínez, María G. Pérez-García, Karla Juarez-Moreno, Josué D. Mota-Morales

**Affiliations:** † Centro Universitario de Ciencias Exactas e Ingenierıas, 42566 Universidad de Guadalajara, Guadalajara, Jalisco 44430, Mexico; ‡ Universidad Nacional Autónoma de México, Centro de Física Aplicada y Tecnología Avanzada, Querétaro, Querétaro 76230, Mexico; § Department of Chemistry and Biochemistry, 2647 The Ohio State University, Columbus, Ohio 43210, United States; ∥ SECIHTI-Instituto de Física, Benemérita Universidad Autónoma de Puebla, Ciudad Universitaria, Puebla 72570, Mexico; ⊥ Centro Universitario de Tonalá, Universidad de Guadalajara, Tonalá, Jalisco 45425, Mexico

## Abstract

Cellulose nanocrystals (CNCs) are promising sustainable nanomaterials, yet their application in biointerfaces is often limited by fixed surface chemistries and limited insight into their concentration-dependent behavior. Here, we report a green and scalable strategy for CNC surface reprogramming using an oxalic acid–choline chloride deep eutectic solvent (ChCl-OAD DES), enabling controlled substitution of sulfate groups with carboxyl functionalities under mild conditions. The resulting carboxylated CNCs (CNC–COOH) preserve crystallinity and morphology while achieving tunable surface charge densities (up to ∼0.19 mequiv g^–1^) and improved thermal stability. We systematically investigate two distinct concentration regimes: dilute dispersions below 0.4 wt %, where CNC–COOH behaves as stable colloids, and concentrated systems at 2 wt %, where percolated hydrogel networks are formed. This transition is governed by hydrogen bonding and ionic screening, leading to pronounced changes in nanoscale organization and viscoelastic behavior. In biologically relevant media, CNC–COOH forms soft, elastic hydrogels (*G*′ ≈ 10^2^ Pa) capable of supporting three-dimensional (3D) cell encapsulation. Importantly, cytocompatibility is strongly dependent on material state. In the colloidal regime (<0.4 wt %), CNC–COOH exhibits negligible cytotoxicity toward 3T3-L1 fibroblasts and weak to mild cytotoxic effects toward HT-29 epithelial cells. In contrast, hydrogel networks (2 wt %) promote high cell viability (>85–100%) and enable 3D cellular organization. Protein adsorption appears to be limited at the surface of the CNC–COOH hydrogel, as indicated by BSA studies, suggesting that ionic strength-mediated interactions play an important role in network formation under cell culture conditions. These findings establish direct correlations among sustainable surface modification, concentration-dependent assembly, and biological response, providing design principles for CNC-based nanomaterials in biointerfaces, 3D cell culture, and nanomedicine.

## Graphical abstract

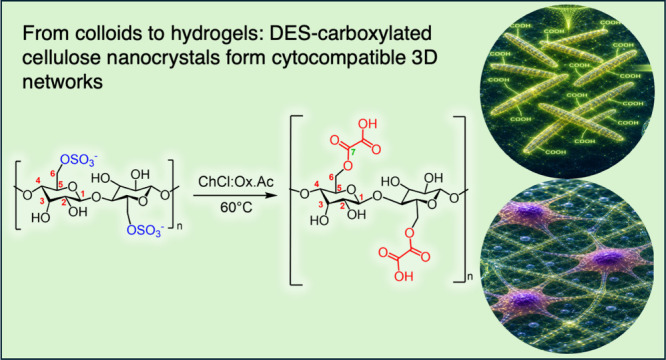

## Introduction

Cellulose is a linear homopolymer composed of β-1,4-linked anhydro-d-glucose units, with molecular weights ranging from approximately 5 × 10^5^ to 2.25 × 10^6^ g mol^–1^ depending on its source and processing history.^1^ Among cellulose-derived nanomaterials, cellulose nanocrystals (CNCs) have emerged as one of the most important building blocks for biobased and biodegradable materials due to their high crystallinity, anisotropic morphology, and versatile surface chemistry. Notably, CNCs have transitioned from laboratory-scale materials to commercially available products, with industrial-scale production achieved by companies such as CelluForce Inc. and Alberta Innovates Technology Futures (Canada), and Borregaard AS (Norway), underscoring their technological maturity and relevance for real-world applications.^2,3^

Industrial CNC production is commonly achieved through sulfuric acid hydrolysis of bleached wood pulp,^2^ whereby the amorphous domains of cellulose fibers are selectively removed, releasing highly crystalline nanorods. This process introduces sulfate half-ester groups onto the CNC surface, conferring colloidal stability in aqueous media but also imparting a fixed surface chemistry that may limit broader functionality.^4,5^ Nevertheless, the presence of abundant hydroxyl and sulfate groups renders CNCs highly amenable to chemical modification, enabling the introduction of diverse functional groups or polymeric moieties through esterification, oxidation, grafting, or adsorption strategies.^1^ As a result, CNCs have been explored in applications ranging from polymer nanocomposites and advanced coatings to hydrogels, drug delivery systems, and biomedical platforms.^6–9^

For biomedical and nanomedicine applications, surface chemistry plays a decisive role in governing CNC interactions with proteins, cells, and biological fluids.^10^ Carboxylated CNCs, in particular, are attractive due to their enhanced colloidal stability, potential for further bioconjugation, and ability to form percolated networks, bioinks, composite biomaterials and hydrogels.^11^ However, conventional carboxylation strategies, such as TEMPO-mediated oxidation or acylation reactions, typically rely on aggressive reagents and multistep purification processes, raising concerns regarding environmental impact, scalability, and cytocompatibility.^12,13^ These limitations have motivated the development of milder and more sustainable functionalization routes that better align with the bioderived and potentially biodegradable nature of CNCs, especially when biomedical or pharmaceutical use is envisioned.^14,15^

In this context, deep eutectic solvents (DESs) have emerged as a promising class of designer solvents, demonstrating strong potential for cellulose processing and surface modification. DESs are composed of hydrogen bond donors and acceptors that form eutectic mixtures with melting points significantly lower than those of the individual components, resulting in liquids at or near room temperature.^16,17^ Their compositional tunability enables precise control over physicochemical properties, including polarity, volatility, degradability, and biocompatibility. Choline chloride (ChCl)–based DESs incorporating carboxylic acids as hydrogen bond donors have been shown to effectively promote CNC esterification or carboxylation under relatively mild conditions, while preserving crystallinity and achieving high yields.^18–21^

The use of DES not only facilitates the production of CNCs but also introduces key functional groups that significantly enhance the properties of the resulting materials,^22^ ranging from emulsion stability to the formation of gelled and porous structures.^23,24^ The ability of DES to reduce environmental impact by lowering energy consumption^25^ and byproduct generation,^26^ while also improving CNC functionality, positions this technology as a crucial component in the development of next-generation, advanced, sustainable materials.

Beyond chemical functionality, CNC concentration in aqueous dispersions critically influences material behavior within the context of biomedicine; surface charge density and particle concentration can profoundly alter CNC–cell interactions, protein adsorption, and local microenvironments, potentially leading to concentration-dependent biological responses.^27^ Moreover, increasing CNC concentration drives a transition from dilute colloidal dispersions to interconnected networks and self-supporting hydrogels, accompanied by pronounced changes in rheological and microstructural properties.^28^ Despite the relevance of these transitions for soft biomaterials and cell-interfacing systems, cytocompatibility is often assessed at a single concentration regime, overlooking the fact that identical CNCs may elicit distinct cellular responses depending on their concentration and material state.^29^

In this work, we present a systematic investigation of the functionalization of CNCs using a DES composed of an equimolar mixture of ChCl and OAD at mild temperatures (60 °C). This work aims to establish a unified relationship between CNC surface chemistry, concentration-dependent assembly, and cytocompatibility across both colloidal and hydrogel regimes. To this end, we examine the concentration-dependent evolution of physicochemical, colloidal, and rheological properties from colloids to hydrogels and directly correlate these parameters with *in vitro* cytocompatibility using murine 3T3-L1 fibroblasts and human HT-29 colorectal adenocarcinoma cells. By correlating sustainable surface modification strategies with material state and biological response, this study establishes design guidelines for DES-derived carboxylated CNCs for 3D cell culture and, more broadly, for potential applications in nanomedicine and biocompatible soft matter systems.

## Experimental Section

### Materials

Choline chloride (ChCl, ≥ 98%, Sigma-Aldrich, BioReagent); Oxalic acid dihydrate (OAD, ≥ 99%, Sigma-Aldrich, ACS reagent); Cellulose nanocrystals CNC, CelluForce Inc., Canada); Ethyl alcohol (≥96%, Golden Bell); deionized water; Dulbecco’s Modified Eagle Medium (DMEM; supplemented with l-glutamine 1%, 1% streptomycin in penicillin, sodium bicarbonate 1.5 g L^–1^, sterile; pH = 7.4); Phosphate Buffered Saline (PBS, 1X, pH = 7.4).

### Preparation of the Deep Eutectic Solvent (DES)

ChCl was first dehydrated for 24 h at 90 °C in a convection oven before use. The DES was prepared by combining equimolar amounts of ChCl and OAD and heating the mixture at 70 °C in an oil bath under constant stirring until homogeneous liquid was formed, following the procedure proposed by Sirviö and col.^30^

### Chemical Modification of the CNC with DES

Figure 1A shows a diagram of the surface modification of CNCs with DES. The CNCs were dispersed in the ChCl-OAD DES (1 wt %) in a hermetic container at a 60 °C using an isothermal bath with magnetic stirring. Samples were taken at 0.5, 1.5, 2, 3, and 4 h of reaction time. The samples were washed seven times using centrifugation with ethanol at 5000 rpm for 10 min. The functionalized and washed CNCs were redispersed in ethanol and stored at 4 °C. The obtained CNCs were used for all experiments.

**Figure 1:**
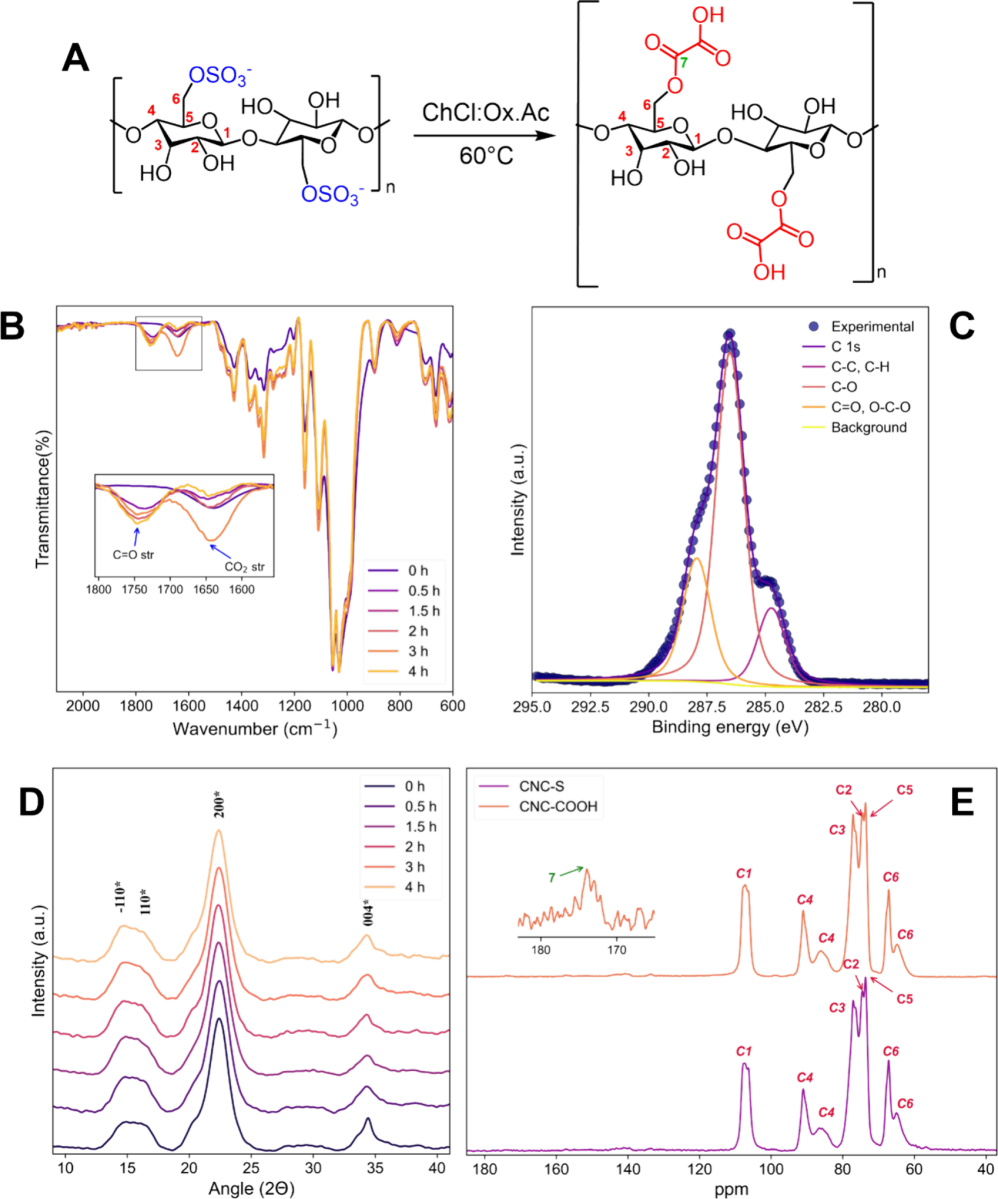
(A) General scheme for the functionalization of CNC-S in ChCl-OAD DES. (B) FTIR-ATR analysis for functionalized CNC, scan from 2000 to 600 cm^–1^, and close-up for carbonyl groups at 1750 cm^–1^ in the inset. (C) High-resolution XPS spectra of CNC at 3 h for C *1s*. Graph description: C *1s*, experimental points (navy blue dots), C *1s* (purple line), C–C, C–H (magenta line), C–O (red line), C=O, O–C–O (orange line), and background (yellow line). (D) XRD analysis of functionalized CNC for reaction times from 0 to 4 h. (E) ^13^C solid-state NMR spectra of CNC–COOH at 3 h (top) and commercial CNC-S (bottom).

### Preparation of CNC-Based Hydrogels

Hydrogels were prepared from functionalized CNC in ethanol. The ethanol-CNC solution was first centrifuged for 10 min at 5,000 rpm, to remove the supernatant, and the wet mass was weighted using a precision scale (previous equivalencies between dried and wet mass were made). Deionized water was then added to achieve a final concentration of 2 wt % of functionalized CNC. Finally, after vigorous stirring to disperse the CNCs, the hydrogels were left to swell for 10 min. Similarly, hydrogels were prepared using a 1:1 mixture of culture Dulbecco’s Modified Eagle’s Medium (DMEM, supplemented with 1% l-glutamine, 1% streptomycin/penicillin, and 1.5 g/L sodium bicarbonate; sterilized and adjusted to pH 7.4) and water to simulate a cell culture environment.

### Fourier Transform Infrared Spectroscopy (FTIR)

FTIR spectra were recorded using a Nicolet iS50 spectrometer (ThermoFisher) with a diamond attenuated total reflectance (ATR) attachment. The data were recorded over the range of 4000–400 cm^–1^ in absorbance mode with 32 scans per spectrum and with a resolution of 0.5 cm^–1^.

### Solid State ^13^C NMR Spectroscopy

Solid State ^13^C NMR were conducted using at 600 MHz on a Bruker AVIIHD NMR spectrometer equipped with a 3.2 mm HXY probe tuned to ^1^H–^13^C double mode. Samples were packed into 3.2 mm zirconium rotors and spun at 10 kHz MAS, and 300 K. Pulses and chemical shifts were externally calibrated to N-acetyl-valine (NAV). ^1^H–^13^C CPMAS experiments were collected with 20k scans each and recycled delays set to 3s.

### X-ray Photoelectron Spectroscopy (XPS)

CNCs were analyzed using a monochromatic Al Kα source (1486.7 eV) operated at 250 W and 12.5 kV, equipped with a 1D DLD detector and a Phoibos 150 analyzer. Functionalized and freeze-dried CNC samples were examined by survey scans and high-resolution scans of the C 1s, O 1s, and S 2p regions. Peak areas and deconvolution analyses were performed using AAnalyzer software. Elemental area ratios for each reaction time were calculated relative to those of the original CNC sample.

### X-ray Powder Diffraction (XRD)

XRD analysis was used to study the crystallinity of the modified CNCs. The samples were analyzed using an Empyrean (Malvern Panalytical). XRD patterns were recorded using a Bragg–Brentano geometry and Cu–Kα radiation of 1.5405 Å, an X-ray source with a voltage of 40 kV and a current of 30 mA was used.

### Atomic Force Microscopy (AFM)

To verify the particle dimensions or aggregates present in the CNCs as a result of the functionalization process, the samples were studied using AFM. Samples for time zero and 3 h of reaction were dispersed in deionized water and subsequently deposited on a silicon substrate and allowed to dry at room temperature to obtain a uniform dispersion. A Bruker Atomic Force Microscope (Dimension Edge) was used to examine the surface morphology of the CNCs before and after the incorporation of the carboxyl groups on their surface. The images were obtained in contact mode with a Bruker SL10 probe.

### Conductometric Titration

Conductivity was measured using the conductometer Orion Versa Star22 (ThermoFisher Scientific). Functionalized CNCs were dispersed in water at 10 wt % for conductometric titration of its surface functional groups. Briefly, NaCl (100 mM) was added to increase the conductivity up to a measurable range. The process was followed by the addition of HCl (100 mM), the acid lowers the solutions’ pH and ensures the protonation of carboxylic acids from the CNCs. The CNCs were then titrated by adding NaOH (2 mM) dropwise, measuring the samples’ conductivity. The corrected conductivity at each data point was calculated by eq 1.

1 $$C_{C}=C_{M}\left(\frac{V_{i}+V_{0}}{V_{0}}\right)$$

where C_c_ is the corrected conductivity in μS cm^–1^, C_M_ is the measured conductivity for each data point (μS cm^–1^), V_i_ is the initial suspension volume in mL (i.e., the total volume of the diluted CNC suspension) and V_0_ is the added volume of NaOH at each point (mL).

### Thermogravimetric Analysis (TGA)

TGA was conducted using Discovery TGA (TA Instruments) in a temperature range from 25 to 500 °C with a heating rate of 20 °C min^–1^ under a nitrogen flow rate of 200 mL min^–1^. The degradation temperature was taken at the first derivative peak of the thermogravimetric curve.

### Elemental Analysis

The functionalized CNCs powder for each reaction time was analyzed by elemental analysis to determine the percentage of each element present in CNC on a TruSpec Micro (LECO).

### Rheology

The rheological properties of the hydrogels (2 wt % of CNC, equivalent to 20.4 mg mL^–1^) formed in water and based on the functionalized CNC were assessed with and ARES-G2 TA Instrument rheometer equipped with a 40 mm diameter cone–plate geometry with a 2° angle. Samples were loaded onto the rheometer’s bottom plate, maintaining a gap of approximately 70 μm between the sample and the plate. All measurements were performed at a constant temperature of 37 °C. To determine the linear viscoelastic region (LVR), oscillation strain was set to a range of 0.1 to 100% at a frequency of 1 Hz. A dynamic frequency sweep test was performed from 0.1 to 100 rad s^–1^, to determine the dynamic storage modulus (G’) and loss modulus (G’’) of each hydrogel at a strain rate confirmed to be within the LVR for each hydrogel. For each formulation three repeated measurements were made, and mean values were reported.

### DLS Measurements

Size distribution measurements were performed using a Zetasizer Advanced Pro Blue (Malvern Panalyticals) of CNC-S and CNC–COOH dilutions (0.02, 0.2, 0.4, 0.8, 1.6, 2, and 4 wt %) in water. Samples were prepared by dispersing CNCs in water using a magnetic stirrer (750 rpm) for 15 min at RT and measured immediately. Each sample was measured in triplicate at room temperature, allowing 0 s of calibration time and 0 s pause between repeats.

### Cell Viability Assay

HT-29 and 3T3-L1 cells were expanded in Dulbecco’s Modified Eagle Media (DMEM) supplemented with 10% fetal bovine serum (B-Bio, Mexico) for HT-29 cells or calf serum (Biowest, FL, USA) for 3T3-L1 cells, 1% l-glutamine, 1% penicillin–streptomycin, and 2.2 g/L sodium bicarbonate. Before cell seeding, hydrogels were disinfected under UV light for 15 min on each side.

Cell viability was evaluated using the MTT (3-(4,5-dimethylthiazol-2-yl)-2,5-diphenyltetrazolium bromide) assay. 3T3-L1 or HT-29 cells were seeded in 96-well plates at a density of 10,000 cells per well in 100 μL of DMEM supplemented and incubated for 24 h at 37 °C in a 5% CO_2_-humidified atmosphere to allow cell attachment.

Freeze-dried CNC samples were resuspended in DMEM at concentrations of 0.25, 0.5, 1, 1.5, 2, and 4 mg mL^–1^. Suspensions were sonicated in an ultrasonic bath for 10 min immediately before use to ensure homogeneous dispersion.

After the initial 24 h incubation, the culture medium was carefully removed, and 100 μL of each CNC suspension was added to the corresponding well. Positive control wells received 100 μL of DMEM, and negative control wells received 20% (v/v) Tween-20 in DMEM (to induce 100% cytotoxicity). Plates were then incubated for an additional 24 h at 37 °C and 5% CO_2_.

Following treatment, the medium was aspirated, and wells were washed twice with PBS. Subsequently, MTT and DMEM were added to each well according to the manufacturer (Sigma-Aldrich, USA). Wells were gently mixed, and the plate was incubated for 4 h at 37 °C in 5% CO_2_ to allow formazan crystal formation. After this, 100 μL of isopropanol was added to each well to solubilize the formazan crystals. Wells were mixed several times, and the plate was incubated in the dark at room temperature for 30 min. Absorbance was measured at 595 nm using a microplate reader (iMark, Bio-Rad, USA). Cell viability was expressed as a percentage relative to the untreated cells (100% cell viability). All experiments were performed in triplicate, and the entire procedure was repeated independently for both 3T3-L1 and HT-29 cell lines.

### Cell Proliferation Assay

Cell metabolic activity, used as an indirect indicator of proliferation, was assessed using resazurin. Hydrogels were exposed to UV-light for 15 min on each side prior to cell seeding. After sterilization, the scaffolds were presoaked in culture medium for 1 h to reach hydration equilibrium. Then, cells were seeded onto each scaffold at a density of 50,000 cells per well, followed by the addition of 1 mL of medium to ensure full immersion. Samples were incubated at 37 °C with 5% CO_2_ for 24 h. After this, the scaffolds were transferred to new wells and incubated in 1 mL of fresh medium for an additional 24 h. Afterward, the medium was replaced with a resazurin-supplemented medium (10% v/v). After a 4-h incubation at 37 °C, absorbance was measured at 570 and 600 nm using a microplate spectrophotometer. A calibration curve was used to estimate cell numbers, and cultures grown without scaffolds served as experimental controls. Cell proliferation was calculated using Equation 2, which determines the percentage reduction of resazurin:

2 $$\mathrm{resazurin reduction (\%)}=\left(\frac{{\mathrm{Eoxi}}_{600}A_{570}-{\mathrm{Eoxi}}_{570}A_{600}}{{\mathrm{Ered}}_{570}C_{600}-{\mathrm{Ered}}_{600}C_{570}}\right)\times 10\%$$

where Eoxi_600_ is the extinction coefficient of oxidized resazurin at 600 nm (117,216), A_570_ is the absorbance of test wells at 570 nm, Eoxi_570_ is the extinction coefficient of oxidized resazurin at 570 nm (80,586), A_600_ is the absorbance of test wells at 600 nm, Ered_570_ is the extinction coefficient of reduced resazurin at 570 nm (155,677), C_600_ is the absorbance of the negative control at 600 nm, Ered_600_ is the extinction coefficient of reduced resazurin at 600 nm (14,652), and C_570_ is the absorbance of the negative control at 570 nm.

### Confocal Microscopy

CNCs were stained with calcofluor white for visualization, whereas 3T3-L1 fibroblasts were labeled with propidium iodide. Calcofluor white is a fluorescent dye with a high affinity for ordered polysaccharides, such as cellulose present in CNCs, and adsorbs to their surfaces in an oriented manner.^31^ Propidium iodide intercalates stoichiometrically into nucleic acids, making fluorescence intensity directly proportional to cellular DNA content.

First, a 1 mL aliquot of CNC-3h suspension (4 wt %) was mixed for 1 min to form a nonflowing hydrogel. Then, 500 μL of this hydrogel was carefully transferred into a glass-bottom confocal dish and spread evenly to cover the entire glass surface. Subsequently, 160,000 3T3-L1 cells suspended in 200 μL of DMEM were gently seeded onto the hydrogel. The dishes were incubated at 37 °C in 5% CO_2_ atmosphere for 2 h to allow initial cell attachment. Subsequently, 300 μL of DMEM was added slowly, and cultures were maintained under standard conditions (37 °C, 5% CO_2_, humidified atmosphere) for a total of 48 h.

After 48 h, the culture medium was removed, and cells were fixed by adding 1 mL of 4% paraformaldehyde in PBS for 15 min at 4 °C, followed by two washes with PBS. Cells were then permeabilized with 0.1% Triton X-100 in PBS for 15 min at 4 °C, followed by two washes with PBS. Cellulose nanocrystals were stained with 50 μL of propidium iodide (1 μg mL^–1^) for 15 min at room temperature in the dark. Finally, samples were washed three times with PBS to remove unbound dye and maintained in PBS until imaging.

### Quantification of BSA Adsorption on CNC Hydrogels Using the BCA Assay

Total protein concentration was determined using the bicinchoninic acid (BCA) assay, a colorimetric method based on the Biuret reaction followed by specific detection of Cu^+^ ions. The assay was performed following the manufacturer’s microplate protocol (Pierce, BCA Protein Assay Kit, Thermo Scientific, MA, USA). A stock solution of 2 mg mL^–1^ bovine serum albumin (BSA) was prepared in Milli-Q water and stored at 4 °C. Serial dilutions of BSA were used to generate a standard curve with final concentrations from 25 to 2000 μg mL^–1^. Triplicate aliquots of standards and samples were loaded into a 96-well plate. Working reagent was added to each well as indicated, and the plate was incubated at 37 °C for 30 min. Absorbance was measured at 562 nm using a microplate reader (BioTek Synergy H, Agilent, CA, USA). Protein concentration in the samples was determined by interpolation from the BSA standard curve using four-parameter logistic regression.

To determine BSA adsorption onto the hydrogels, a 2% (w v^–1^) CNC-3h hydrogel was prepared, and BSA was incorporated at a final concentration of 250 μg mL^–1^. The mixture was gently homogenized and incubated at 37 °C for 48 h to allow protein adsorption. After incubation, the hydrogel was centrifuged at 5,000 g for 5 min to sediment the CNC network. An aliquot of the suspension was taken to determine the amount of BSA using the BCA method. The amount of adsorbed BSA was calculated by subtracting the protein concentration remaining in the supernatant from the initial concentration, using a BSA standard curve prepared under identical conditions.

The statistical analysis carried out was a two-way ANOVA with Dunnett’s multiple comparisons posthoc test using GraphPad Prism.

## Results and Discussion

The surface modification of commercial CNCs, which naturally contain sulfate groups (CNC-S), was carried out following established protocols developed by our group.^24,32,33^ In this study, a CNC-S concentration of 1 wt % in the ChCl-OAD DES was selected, as higher concentrations led to gelation of the colloid. Surface functionalization via the introduction of carbonyl groups was achieved under mild temperature conditions (60 °C, Figure 1A) in the DES and was initially quantified by conductometric titration, as discussed below. Samples were collected at 0.5, 1.5, 2, 3, and 4 h of reaction, thoroughly rinsed with ethanol to remove the DES, and further analyzed using multiple characterization techniques.

Carbonyl groups were quantified by aqueous conductometric titrations according to the procedure described by Foster et al.^34^ As shown in Figure S1 (SI), the amount of milliequivalents per gram of CNC increases with reaction time. The sulfur content in commercial CNCs reported by the supplier CelluForce (0.102 to 0.183 mequiv gCNC^–1^) is consistent with the value obtained in this study (0.145 mequiv gCNC^–1^, Table S1, SI).

FTIR spectroscopic characterization of dried CNC samples collected at different functionalization times in ChCl-OAD DES revealed the progressive development of absorption bands associated with carboxylic acid functionalities (Figure 1B). The typical cellulose peaks corresponding to the stretching vibration of the glycosidic bond at 1164 cm^–1^, the carbon–oxygen bond at 1113 cm^–1^, the pyranose ring backbone at 1059 cm^–1^, and the carbonyl groups at approximately 1725 cm^–1^ are observed in the spectra.^35,36^ The intensity of the signal corresponding to the carbonyl groups (vibration of the C = O bond) increases as the reaction time advances, while the signal from the pyranose ring remains constant, pointing to the CNC surface functionalization.

Figure 1B also shows that the signal of carbonyl groups increases significantly after 1.5 h of reaction, while at 2 and 4 h, it increases more subtly. This suggests not only that the surface functionalization is effective, and the primary structure of CNC remains unchanged, but also that the reaction kinetics of CNC carboxylation are faster at the beginning and slow down as the reaction progresses.

The esterification of CNC with oxalic acid was confirmed with the ^13^C solid-state NMR spectroscopy, as shown in Figure 1E. The spectrum of functionalized CNC (CNC–COOH, at 3h of functionalization) exhibited carbon peaks characteristic of cellulose I, specifically *C1* at 107.53 ppm, *C4* at 91.10 and 86.30 ppm, and *C6* at 67.18 and 64.94 ppm. The signals for *C4* and *C6* appeared in two distinct regions, in accordance with the presence of amorphous nonhydrolyzed cellulose chains (at high frequencies) located on the surface, as well as the signal from the crystalline part (at low frequencies). Additionally, the *C2*, *C3*, *C5* cluster yielded peaks at 74.52, 76.88, and 73.71 ppm, respectively. Moreover, a small *C7* peak is observed around 174 ppm, attributed to the resonance of carbonyl in ester carbon, indicating the successful introduction of ester groups onto the CNC surface after carboxylation. These signals are consistent with those reported by other authors for the functionalization of CNC with oxalic acid.^37,38^ To gain quantitative insights into the surface chemical composition of CNC–COOH, the samples were analyzed by XPS to determine the main elements (O *1s*, C *1s* and S *2p*) (Figure S2A, SI), and to monitor their temporal evolution as the functionalization progressed. Deconvolution of the high-resolution C *1s* spectra for CNC at 3 h of functionalization revealed peaks at 284.6 eV (C–C/C–H), 286.6 eV (C–O), and 287.6 eV (C = O), (Figure 1C). XPS spectra for C *1s* at different functionalization times can be found in the SI.^39,40^ In addition, the high-resolution S *2p* spectra were fitted in an analogous manner to the C *1s* region, allowing the area of each component peak to be determined (Figure S2, SI). The ratio between the S *2p* and C *1s* signal areas was plotted as a function of reaction time (Figure S3, SI). A progressive decrease in the S/C ratio was observed with increasing reaction time, in agreement with the conductometric titration and FTIR results. The calculated S *2p* and C *1s* peak areas are summarized in Table 1. As the reaction proceeded, the S *2p* area decreased while the C *1s* area increased, confirming the effective substitution of sulfate groups by carbonyl functionalities. Similarly, the elemental analysis results reveal clear compositional changes between the original (CNC-S) and functionalized CNC–COOH (Figure S4, SI). In particular, the carbon and hydrogen contents increase, whereas the sulfur and oxygen contents decrease upon functionalization. These trends further support the substitution of sulfate groups by carbonyl functionalities. The decrease in oxygen is not proportional to that of sulfur, which is expected given that sulfate groups contain multiple oxygen atoms per sulfur atom.

**Table 1:** S *2p* and C *1s* Areas and S/C Area Ratios of Peaks Resulting from XPS Analysis of Functionalized CNC

reaction time (h)	S *2p* area (a.u.)	C *1s* area (a.u.)	S/C (X1000)
0	265.1	24534.3	10.81
0.5	308.7	34940.3	8.83
1.5	197.9	35051.0	5.65
2	125.0	28851.0	4.33
3	222.2	33707.3	6.59
4	129.2	35499.1	3.64

To confirm that functionalization occurred selectively on the CNC surface, the crystallinity of the samples was evaluated by XRD. X-ray diffractograms (Figure 1D) display characteristic diffraction peaks at 2θ = 14.8°, 16.6°, 22.8°, and 34.6°, which correspond to the (−110), (110), (200), and (004) crystallographic planes of CNC, respectively. A crystallinity index of 97% for 0 h (CNC-S) and 96% for 4 h was calculated using the Segal equation, which gives the relative crystallinity index comparing the intensities at 18 (*I*_*am*_) and 22.8 2θ (*I*_*200*_), corresponding to amorphous cellulose and plane (200), respectively (eq 3). These results indicate that the functionalization process does not significantly affect CNC crystallinity, even after 4 h at 60 °C, further confirming that the introduction of carboxylic acid groups occurs primarily at the CNC surface.^41^ This is consistent with the highly crystalline nature of CNCs, which contain negligible amorphous regions, and with the fact that the DES does not disrupt the hydrogen-bonding network within the crystalline domains at the mild reaction conditions.^42^

3 $$\mathrm{CI}=\frac{I_{200}-I_{\mathrm{am}}}{I_{200}}\times 100$$

Further analysis of the effect of surface functionalization on CNC dimensions and morphology was performed by AFM on CNC-S and CNC–COOH at 3 h.

AFM images presented in Figure 2 show that even after 3 h (Figure 2B) of functionalization using a ChCl-OAD DES, the CNC maintains its needle-like morphology, exhibiting no apparent rupture, nor significant decrease in dimensions. From the AFM images of individual nanocrystals (Figure S5, SI), CNC–COOH functionalized for 3 h exhibited an average length of approximately 207 nm, whereas the as-purchased CNC-S (0 h; Figure 2A) measured about 208 nm. These results indicate that neither the DES nor the functionalization temperature significantly affects CNC morphology, supporting the surface-specific introduction of carboxylic groups without cellulose hydrolysis or loss of crystallinity, consistent with the calculated Segal crystallinity index.

**Figure 2:**
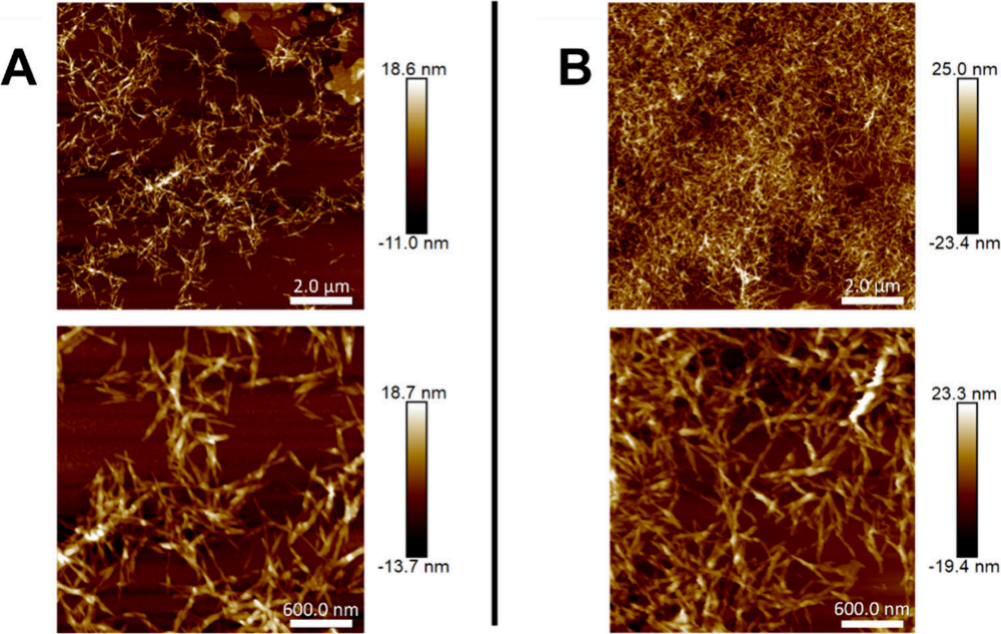
AFM images taken using contact mode of (A) CNC-S before DES treatment (0 h) and (B) CNC–COOH after functionalization for 3 h using DES.

From a thermal standpoint, TGA analysis (Figure S6, SI) indicates that the decomposition temperature of CNC–COOH increases upon functionalization. Overall, this improved thermal stability can be attributed to the introduction of carboxyl groups.^41^ The onset temperature of the main decomposition step rises from 308 °C for the commercial CNC-S to 333 °C after 4 h of DES treatment in CNC–COOH, which can be attributed to half-ester sulfate groups acting as catalytic sites for dehydration while their replacement by more stable organic groups prevents early carbonization, thereby increasing the decomposition temperature.^43,44^ In addition, the lower residual mass observed for CNC–COOH is likely due to the substitution of inorganic sulfate groups.^45,46^

To better understand the behavior of the CNC dispersed in water, i.e. in its aqueous colloidal state, DLS measurements were performed in commercial cellulose (CNC-S) and functionalized cellulose (CNC–COOH) obtained at 3h of reaction, at different concentrations (0.02, 0.2, 0.4, 0.8, 1.6, 2, and 4 wt %), DLS distributions can be found in the SI. As shown in Figure 3A, CNC-S sample exhibits an exponential increase in apparent size with increasing concentration, from 192 to 1534 nm. At low concentrations (<0.8 wt %), individual nanocrystals behave as dispersed colloids; however, as the concentration increases, the crystals progressively self-assemble into larger aggregates, ultimately forming stable 3D networks (i.e., hydrogels).^47^ This behavior arises from the concentration- and ionic-strength-dependent stability of CNC rod-like particles. At low ionic strength, electrostatic repulsion dominates, leading to stable dispersions. In contrast, at higher ionic strength the electrical double layer becomes compressed, attractive interactions prevail, and gel formation is promoted.^48^ Similar results are observed for CNC–COOH functionalized for 3 h where the size measured increases from 399 to 818 nm; however, this transition to hydrogel aggregates is observed above 2 wt %, whereas colloid-like behavior is constant for concentrations up to 1.6 wt % (Figure S7, SI). This can be attributed to the surface functionalization of DES-treated CNC, as carboxyl functional groups facilitate the attraction between crystals through hydrogen bonds, promoting the formation of hydrogels. Moreover, despite the lower degree of functionalization of the modified CNCs reported in this work in comparison to other surface-modification methodologies,^47,49^ the acid surface of the modified CNCs possesses a high charge density that promotes the formation of a stable colloid and a compact hydrogel at higher concentrations.^11^ Although DLS calculates the hydrodynamic radius based on the Stokes–Einstein equation, which assumes the diffusion of spherical particles, these results consistently reflect the concentration-dependent aggregation behavior of rod-like CNCs and provide a reliable comparative assessment of the colloidal-to-gel transition induced by surface functionalization. A similar trend was reported by Azzam et al.,^47^ for TEMPO-oxidized, carboxyl-functionalized CNCs, where the hydrodynamic radius increased up to 6-fold upon heating the suspensions to 40 °C, indicating enhanced aggregation. This behavior was attributed to the weakening of electrostatic repulsive interactions, which facilitates particle association.

**Figure 3:**
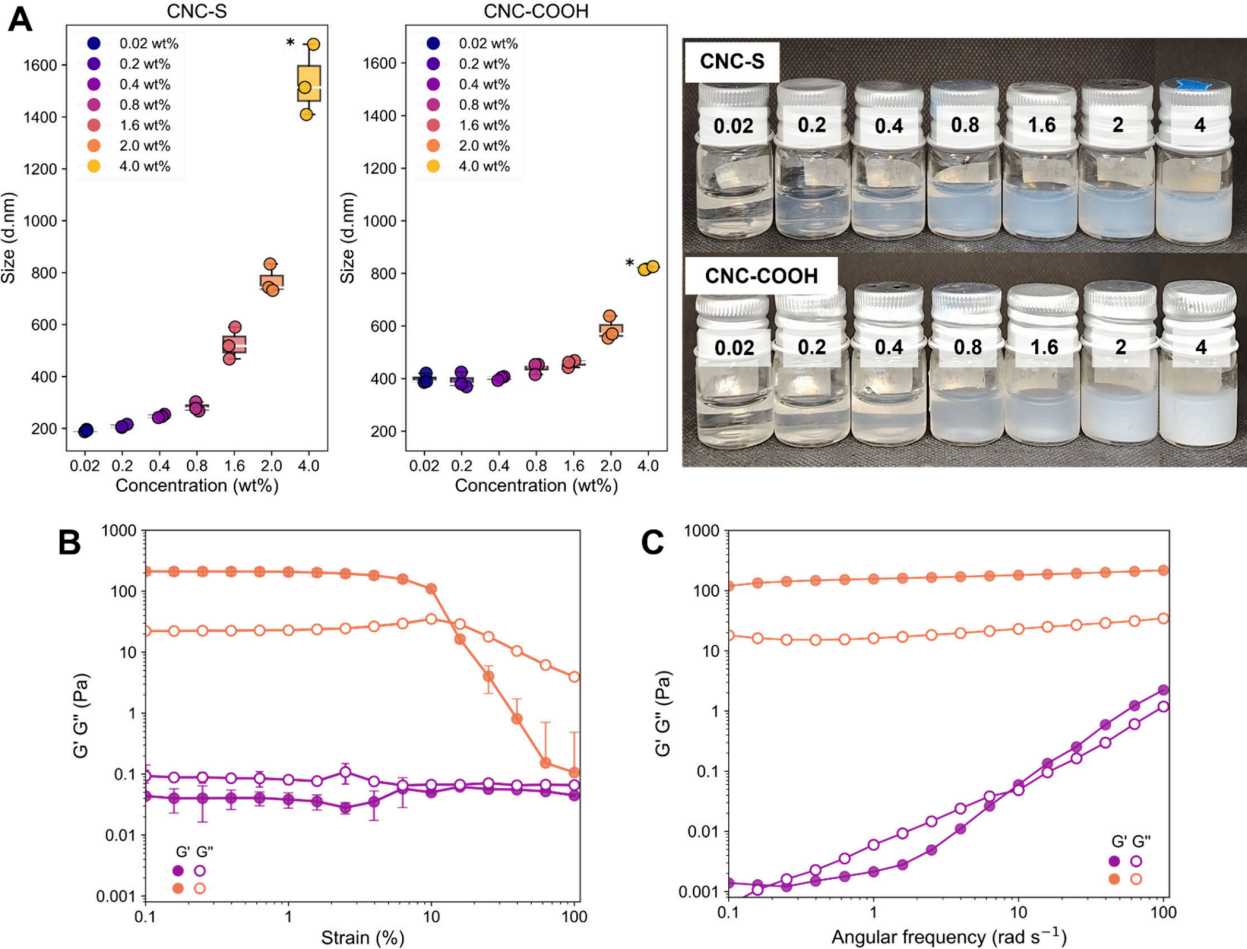
(A) DLS measurements of commercial CNC-S (left) and functionalized CNC–COOH, after functionalization for 3 h (center) at different aqueous concentrations with a digital photograph of the prepared samples (right). (B) Oscillatory amplitude sweep and (C) frequency sweep responses of CNC–COOH hydrogels (2 wt %) prepared in water (purple dots) and DMEM (orange dots). Frequency sweeps were performed at 2% strain for the CNC hydrogel in water and at 10% strain for the CNC hydrogel in DMEM. *Unreliable measurements.

To further understand the esterification of the CNCs, ζ-potential measurements were done on the CNC-S and carboxylated CNC (Figure S8, SI). A dramatic shift in surface charge from −100 mV to −41 mV was observed after DES treatment for 3 h, which suggests high colloidal stability and further confirms the surface functionalization of the crystals. This shift can also be attributed to the degree of deprotonation of the carboxyl groups influencing the overall surface charge.^11,19^

Resulting CNC hydrogels were also rheologically characterized within the linear viscoelastic region (LVR), defined as the deformation range in which the storage and loss moduli (*G*′ and *G*″, respectively) remain independent of strain (γ%). To determine the LVR, strain sweeps were performed from 0.01 to 100% at a constant frequency (*f* = 1 Hz) and temperature of 37 °C. Figure 3B shows the strain-dependent evolution of *G*′ and *G*″ for CNC hydrogels prepared in different media.

The viscoelastic response strongly depended on the preparation medium. For CNC–COOH hydrogels prepared in water (Figure 3B, purple symbols), *G*′ remained close to *G*″ across the entire strain range, with *G*″ consistently exceeding *G*′. Both moduli exhibited very low absolute values (0.01–0.3 Pa), indicating weak internal cohesion and the absence of a well-developed percolated network.^50^ In addition, *G*′ and *G*″ remained nearly constant up to γ ≈ 2%, defining the LVR, after which both moduli gradually decreased without a clear crossover point (*G*′ = *G*″). Overall, these results indicate that carboxylated CNC dispersions in water behave as weakly structured viscoelastic fluids, consistent with the colloid-like behavior observed by DLS.^51^

To better understand the behavior of CNC–COOH under biologically relevant conditions, rheological tests were also performed in DMEM, the medium used for cell culture and viability experiments. In contrast to water, carboxylated CNC hydrogels prepared in DMEM (Figure 3B, orange dots) exhibited a predominantly solid-like response, with *G*′ remaining approximately 1 order of magnitude higher than *G*″ within the low-strain region (0.1–10%). In this range, *G*′ values of ∼100–200 Pa and *G*″ values of ∼10–20 Pa confirm the formation of an elastic network. Beyond γ ≈ 10%, both moduli decreased with increasing strain, indicating the onset of yielding and departure from the LVR. At larger deformations, *G*′ progressively decreased while *G*″ increased, eventually leading to *G*″ surpassing *G*′, consistent with a transition toward more liquid-like behavior under high strain.

Consistent with previous reports describing substantial CNC assembly transitions at ionic strengths of approximately 0.05–0.2 M^48^), the ionic strength of DMEM (∼0.15–0.18 M)^52,53^ promotes electrostatic screening of negatively charged CNC–COOH surfaces, resulting in a transition from weakly structured viscoelastic behavior in water to percolated hydrogel networks, as evidenced by the evolution from *G*′ values comparable to *G*″ in water to a more than one-order-of-magnitude difference in DMEM (*G*′ ≈ 200 Pa vs *G*″ ≈ 20 Pa).

The enhanced elasticity in DMEM is consistent with the aggregation state identified by DLS, which showed increased hydrodynamic size and CNC clustering in this aqueous media.^54,55^ This behavior is also in agreement with previous reports showing that increasing ionic strength promotes CNC gelation by screening electrostatic repulsion and facilitating attractive interparticle interactions, leading to stronger networks that yield at lower strains. A similar mechanism likely operates in DMEM-based hydrogels, where ionic species (e.g., Na^+^, K^+^, Ca^2+^, Mg^2+^, and phosphate-containing buffers) reduce CNC–CNC electrostatic repulsion and promote the formation of a percolated network.^56^ Overall, these results demonstrate that DMEM promotes the formation of mechanically robust carboxylated CNC hydrogels compared with water, producing higher elasticity at low deformation and earlier yielding under increasing strain^49,57–59^

Figure 3C shows the frequency dependence of *G*′ and *G*″ for CNC hydrogels prepared in water and DMEM. For hydrogels prepared in water (purple dots), *G*″ remained higher than *G*′ across the entire frequency range, and both moduli increased with frequency, indicating a predominantly viscous response with limited structural stability over long time scales. The low magnitude of both moduli, particularly at low frequencies, further supports weak interparticle interactions and transient structuring in aqueous CNC–COOH dispersions, consistent with previous reports for CNC suspensions at concentrations below 10 wt %.^60–63^

In contrast, carboxylated CNC hydrogels prepared in DMEM (orange dots) exhibited *G*′ values consistently higher than *G*″ across the entire frequency range. Both moduli displayed weak frequency dependence, reflecting the formation of a stable internal network typical of physically cross-linked hydrogels. The magnitudes of *G*′ (∼100 Pa) and *G*″ (∼10–20 Pa) further support the presence of a soft solid-like structure dominated by noncovalent CNC–CNC interactions in this ionically enriched medium.^59,60,63^

To assess how CNC functionalized for different durations in the ChCl-OAD DES, thereby yielding varying densities of surface carboxylic acid groups, affects the metabolic activity of HT-29 and 3T3-L1 cells, two CNC concentration regimes were evaluated. The first regime consisted of stable CNC colloids in the range of 0.25–4 mg mL^–1^ (corresponding to ∼0.025–0.40 wt %), which behave as individual nanocrystals (with different surface carboxylic acid densities) and were previously characterized by DLS (Figure 3A). The second regime corresponded to CNC concentrations that promote hydrogel formation (e.g., 2 wt %), prepared in DMEM cell culture medium, enabling the nanocrystals to assemble into a scaffold-like network surrounding the cells; this condition was previously characterized by rheology (Figure 3B–C).

These two cell lines were selected to represent connective (3T3-L1) and nonconnective/epithelial (HT-29) tissue types, respectively. This comparative approach allowed us to examine potential differences in cellular responses across both CNC concentration regimes, particularly considering that connective tissue-derived cells typically rely on strong adhesion to the extracellular matrix to support migration, homeostasis, and long-term survival. Accordingly, HT-29 and 3T3-L1 viability was evaluated using CNC functionalized for different times in ChCl-OAD DES, exhibiting surface carboxylic acid functionalities ranging from 0.145 ± 0.035 mequiv gCNC^–1^ (commercial CNC-S, time 0) to 0.19 ± 0.012 mequiv gCNC^–1^ (CNC–COOH, 3 h), using the MTT assay. It is important to note that within this concentration range, CNCs remain uniformly dispersed in the culture medium as stable colloids due to electrostatic stabilization provided by surface carboxylate groups.

Figure 4A illustrates the viability of HT-29 cells as a function of CNC concentration and the degree of surface functionalization (at different functionalization times), as measured by the MTT-reduction assay. As the CNC concentration increased from 0.25 to 4 mg mL^–1^, cell viability for unfunctionalized CNC-S (0 h) remained between 87.2% ± 6.2% and 90.89% ± 4.07%, respectively. However, at a fixed CNC concentration of 4 mg mL^–1^–a higher concentration that still behaves as stable colloids– cell viability varied with the degree of surface carboxylation (up to a maximum of 0.145 achieved at 3 h of functionalization) as follows: 84.77% ± 3.98 for CNC-1 h; 99.39% ± 7.18 for CNC-2 h, and 92.32% ± 10.4 for CNC- 3 h. Statistical analysis revealed significant differences from the control (*p < 0.05) in many cases. For instance, significant differences were observed in the mean cell viabilities induced by CNC functionalized with carboxylic groups after 2 and 3 h, at CNC–COOH concentrations of 0.25, 0.5, 1, and 1.5 mg mL^–1^, which can be rationalized in terms of their hydrodynamic size as revealed by DLS. Recent evidence shows that the biological response to nanocellulose is strongly dependent on particle morphology and colloidal state, where rod-like CNC can induce different cytocompatibility and immune-cell responses compared with more compact nanocellulose structures, highlighting that aggregation or gelation can modulate cellular outcomes.^64^ Likewise, CNC surface charge and functionalization critically govern cell–nanoparticle interactions, where altered surface chemistry changes cellular association and internalization, and can shift cytotoxicity, emphasizing that even subtle modifications in CNC surface groups can significantly impact viability,^27^ as observed in the present work.

**Figure 4:**
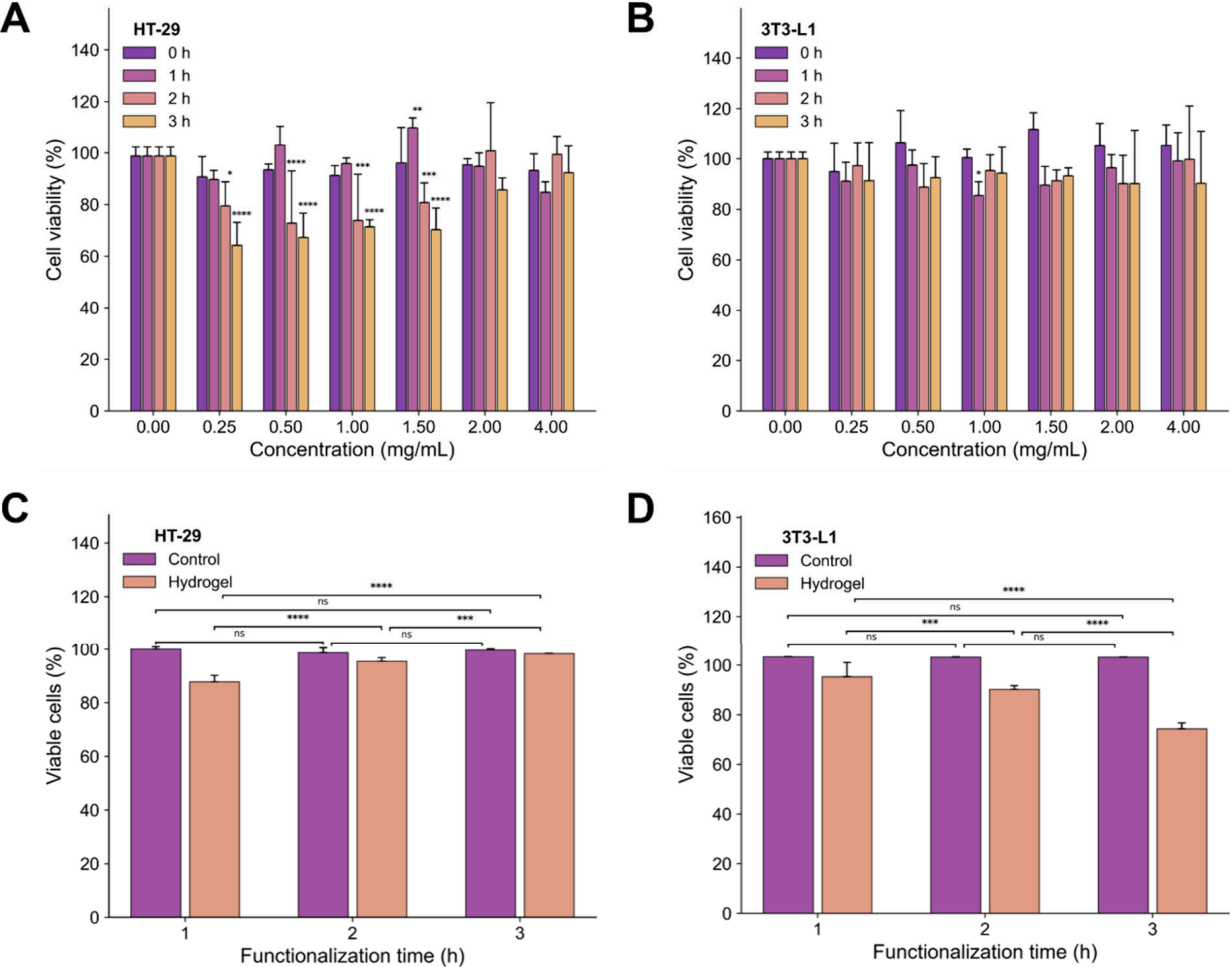
Cell viability assay after incubation for 24 h to evaluate the material toxicity of functionalized CNC at different reaction times and varying concentrations in (A) colon cells (HT-29) and (B) fibroblasts (3T3-L1). Cell proliferation assay after incubation for 48 h to evaluate the biocompatibility of 2 wt % CNC–COOH, i.e., in the form of hydrogels, in (C) colon cells (HT-29) and (D) fibroblasts (3T3-L1). Results are presented as the mean ± SD (*n* = 3). Using two-way ANOVA with a Dunnett’s multiple comparisons posthoc test, *p < 0.05, **p < 0.01, ***p < 0.001, and ****p < 0.0001.

Similarly, Figure 4B depicts the viability of 3T3-L1 cells as a function of CNC concentration as determined by the MTT reduction assay. At a CNC concentration of 4 mg mL^–1^, cell viability remained above 80% for all samples, regardless of the surface carboxylation degree, i.e. different times of functionalization. Statistical analysis showed no significant differences compared to the control (*p* > 0.05). However, a significant difference was observed in the mean cell viability between the 0 h (unfunctionalized) and 1 h samples at a concentration of 1 mg mL^–1^, which may be attributable to particle agglomeration. These results indicate that the presence of surface carboxyl groups on the CNCs has no substantial impact on cell viability in 3T3-L1 fibroblasts. Overall, within the concentration range evaluated (0.25–4 mg mL^–1^), the MTT assays revealed cell-type-dependent responses to CNC exposure. While 3T3-L1 fibroblasts maintained viability comparable to the control across all concentrations tested, HT-29 cells exhibited statistically significant but moderate reductions in metabolic activity. Importantly, viability remained above 75% in all cases, indicating overall low cytotoxicity within the evaluated concentration range.

The MTT-reduction assays further demonstrated that all CNC samples, across the concentration range of 0.25 to 4 mg mL^–1^, exhibited minimal cytotoxicity toward both HT-29 and 3T3-L1 cells. It is worthy to note that direct resuspension of CNCs bearing surface carboxylic groups in DMEM initially produced an acidic pH (pH 4), which is incompatible with cell culture, as evidenced by the color shift of the phenol red indicator (Figure S9, **SI**). To address this issue, all CNC samples were thoroughly washed with PBS before final dispersion in complete DMEM, thereby ensuring physiological pH and optimal culture conditions.

A second approach was designed to investigate the effect of hydrogels composed of CNC functionalized for 1, 2, and 3 h of reaction on the cellular response of HT-29 and 3T3-L1 cells. In this case, CNC–COOH samples were evaluated at a fixed concentration of 2 wt % (∼20 mg mL^–1^), where hydrogel formation occurs. Note that commercial CNC-S was unable to sustain the formation of stable hydrogels in DMEM and was therefore not included in this part of the study. This strategy enables direct assessment of cell viability upon contact with scaffold-like structures composed of CNC–COOH, similar to what has been reported for bacterial nanocellulose hydrogels.^31^

Several studies have evaluated the cytotoxicity of cellulose-based scaffolds toward HT-29 cells. For instance, Cacicedo and col.^65^ reported a marked reduction in cell viability from 95% to 53% when HT-29 cells were cultured on bacterial cellulose scaffolds exposed to doxorubicin at concentrations below 500 μM.

Metabolic activity, which directly correlates with cell proliferation and viability, was assessed using the resazurin reduction assay. A 2 wt % CNC–COOH (∼20 mg mL^–1^) concentration was selected based on prior water-retention and rheological characterization, which demonstrated that this is the minimum concentration required to form a macroscopic, predominantly elastic hydrogel even at high oscillatory frequencies. Cells were encapsulated within the CNC–COOH, hydrogel network throughout the culture period, providing a more physiologically relevant 3D environment than the 2D exposure conditions used in typical MTT assays.

HT-29 cells were cultured for 48 h while fully embedded in 2 wt % CNC–COOH hydrogels. The proportion of metabolically active cells relative to the initial seeded population was 87.8% for CNC–COOH at 1 h, increased to 95.5% for CNC–COOH at 2 h, and reached 98.4% for CNC–COOH at 3 h sample (Figure 4C). These results indicate excellent cytocompatibility and suggest that surface carboxyl groups may enhance cell survival.

These findings compare favorably with previous reports on alternative polysaccharide-based scaffolds for HT-29 cells. Overall, these data highlight CNC hydrogels as an emerging 3D culture platform for HT-29 cells, consistently supporting cell viability above 87% and up to 98.4% even when cells are fully embedded at effective nanomaterial concentrations of 40 mg mL^–1^ forming a percolated network, i.e. a hydrogel.

The results regarding the influence of 2 wt % CNC-hydrogels on 3T3-L1 fibroblasts are presented in Figure 4D, where cell viability was found to exceed 75% for all three types of CNC (CNC–COOH obtained at 1, 2 and 3h of functionalization). Furthermore, the CNC hydrogels effectively retained a high percentage of the cells initially seeded in the culture, with cell viability of 95.3% for CNC–COOH at 1h, 90.2% for CNC–COOH at 2h, and 74.3% for CNC–COOH at 3h.

In this context, Strätz et al. reported that hydrogels composed of oxidized sulfated cellulose exhibited toxicity toward 3T3-L1 fibroblasts at concentrations above 0.5 mg mL^–1^. In contrast, Peschel et al.^66^ found cell viability exceeding 90% when 3T3-L1 fibroblasts were cultured in the presence of sulfated cellulose (1 mg mL^–1^) and above 85% in the presence of carboxymethylated cellulose at the same concentration. Consistent with these previous studies, the viability of 3T3-L1 cells in the presence of cellulose-based materials has generally been shown to be reduced by less than 15%. In our work, we confirm this trend, with no substantial cytotoxicity observed even at CNC concentrations up to ∼20 mg mL^–1^ (2 wt %).

Thus, cell viability in HT-29 and 3T3-L1 cells is directly correlated with metabolic activity, demonstrating that CNC hydrogels at ∼20 mg mL^–1^ maintain viability above 80% for HT-29 cells and above 75% for 3T3-L1 fibroblasts. These results indicate that the hydrogel is biocompatible, in accordance with ISO 10993 for medical devices.

To visualize 3T3-L1 fibroblasts embedded within a 2 wt % (20 mg mL^–1^) CNC–COOH 3h hydrogel, confocal microscopy was employed. Z-stacking imaging spanning approximately 100 μm in the *z*-direction enabled multiplanar analysis. Fibroblasts 3T3-L1 cells were selected for this analysis because their growth and viability in the CNC–COOH hydrogels were highly consistent across all functionalization times (0 to 3 h) and tested concentrations, as demonstrated in the biocompatibility assays. This uniformity allows reliable visualization of cell behavior within a 3D hydrogel matrix independent of the degree of carboxylation.

Serial z-planes illustrate the distribution of cells from the top of the hydrogel to the bottom layer (Figure 5). In the basal plane (Figure 5D), fibroblasts exhibit the characteristic elongated, spindle-shaped morphology typical of adherent cells in 2D culture. In contrast, cells located in upper planes (Figure 5A-C) display rounded morphology with smaller, circular nuclei, indicative of a 3D growth environment within the hydrogel matrix. The merged 3T3-L1/DIC images confirm that these suspended cells adopted a spherical shape markedly distinct from the fusiform morphology of surface-adherent cells. The transition from elongated to rounded cell shape in suspended 3T3-L1 cells within the carboxylated CNC hydrogel closely resembles the morphological changes associated with early adipogenic differentiation, as reported by Mavil-Guerrero et al.^31^ in carboxylated bacterial cellulose scaffolds. Such scaffolds promote the maturation of 3T3-L1 cells into mature adipocytes, characterized by reduced filopodia, a spherical cell body, and lipid droplet accumulation, even in the absence of exogenous differentiation chemical inducers. The similar morphological features observed here suggest that carboxylated CNC hydrogels may provide a favorable 3D environment that facilitates cell maturation and differentiation without additional chemical stimuli.

**Figure 5:**
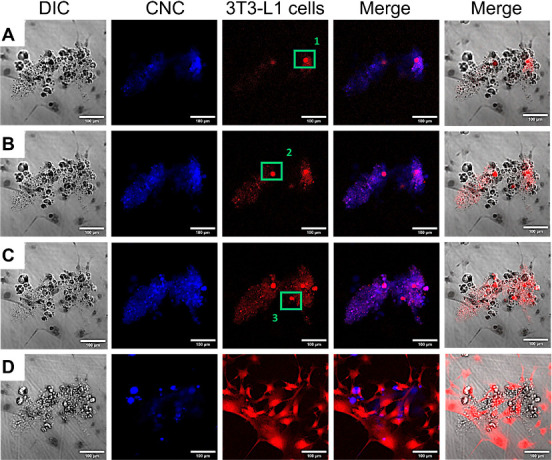
Confocal microscopy images of 3T3-L1 fibroblasts after culturing for 48 h within a 2 wt % carboxylated CNC-3 h hydrogel. The Z-stack reconstruction spans approximately 100 μm in depth, allowing multiplanar analysis. The scale bar was 100 μm. CNC was stained with calcofluor white (blue), and 3T3-L1 fibroblast nuclei were stained with propidium iodide (red).

A lateral (side) view of the 3D reconstruction from the z-stack confocal images reveals a hydrogel thickness of approximately 100 μm (Figure 6A), while Figure 6B clearly identified the individual fibroblasts previously highlighted in Figure 5A-C, surrounded by the CNC matrix. Fibroblasts suspended within 2 wt % CNC–COOH 3 h matrix consistently exhibit a rounded morphology, reflecting 3D growth supported by the CNC–COOH network. This morphological shift is attributed to physical interactions among cellulose scaffolds, likely involving electrostatic forces, hydrogen bonding, and van der Waals interactions, which provide sufficient structural support for cell growth. The observed sphericity is consistent with isotropic contractile forces and cytoskeletal cortical tension beneath the cell membrane of cells grown in hydrated gels, resulting in spherical cell organization without evidence of cell aggregation.^67^

**Figure 6:**
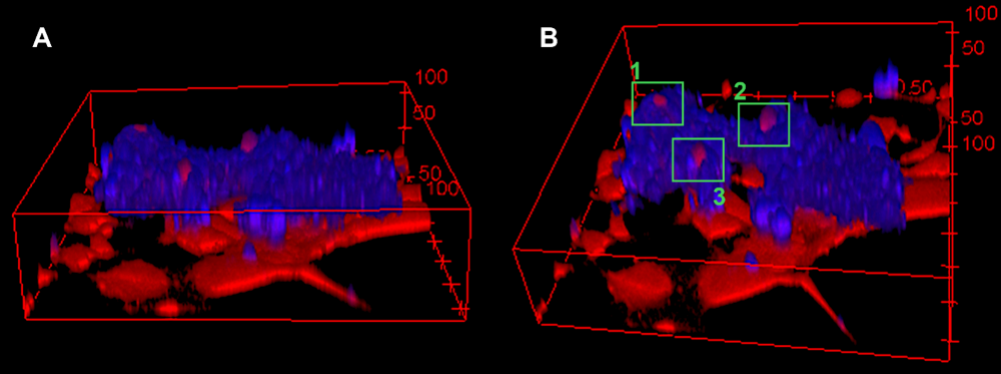
Lateral view of 3D reconstruction of 3T3-L1 fibroblasts after culturing for 48 h within a 2 wt % carboxylated CNC-3 h hydrogel. (A) The z-stack reconstruction spans approximately 100 μm in depth, enabling visualization of the cell distribution throughout the hydrogel thickness. (B) Identification of fibroblasts (red) previously highlighted in Figure 5 (panels A and B), which are fully surrounded by the CNC matrix (blue). Suspended fibroblasts within the 2 wt % CNC-3 h matrix consistently display a rounded morphology, consistent with 3D growth supported by the carboxylated CNC network. CNC was stained with calcofluor white (blue), and 3T3-L1 fibroblast nuclei were stained with propidium iodide (red).

These observations align with previous quantitative data indicating that cell growth exceeds 74.3% within CNC–COOH hydrogels. The confocal images further confirm that, although cells in direct contact with the dish surface retain their typical 2D fusiform morphology, the majority remain uniformly distributed and viable throughout the 3D hydrogel volume.

This short-term viability study demonstrates that carboxylated CNC hydrogels are cytocompatible across distinct cellular models, including 3T3-L1 fibroblasts, which possess adipogenic differentiation potential, and HT-29 epithelial cells, which can develop mucus-producing phenotypes under appropriate conditions and are known to respond to 3D microenvironments.

To disregard whether proteins present in DMEM contributed to CNC aggregation—potentially through interactions with surface carboxyl groups and subsequent protein corona formation—an adsorption study of BSA onto CNC–COOH (3 h) was conducted. After 48 h of incubation at 37 °C, the final BSA concentration in the supernatant was 264 μg mL^–1^, compared to an initial concentration of 250 μg mL^–1^. The slight increase is attributed to minor water loss from the hydrogel during incubation rather than to BSA adsorption.

This observation is consistent with previous studies on cellulose nanocrystals bearing positively charged (e.g., pyridinium) or negatively charged (sulfated and carboxylated) surface groups, which report preferential protein binding to positively charged surfaces.^68^ This shift promotes the transformation of dilute colloidal dispersions into percolated networks and self-supporting hydrogels. These results indicate that DMEM primarily acts as a physicochemical trigger for hydrogel formation through ionic strength–mediated screening of electrostatic repulsion between CNC–COOH particles, whereas the enhanced cytocompatibility observed at 2 wt % arises from the resulting hydrogel architecture and altered cell–material interactions rather than from the ionic composition of the medium alone.

Overall, these findings highlight the importance of surface charge and medium composition in the design of cellulose-based scaffolds, as both parameters critically influence interparticle interactions and potential biointerfacial behavior.

## Conclusions

We present a sustainable and controllable strategy for the surface engineering of CNCs using an oxalic acid–based DES, enabling the selective introduction of carboxyl functionalities while preserving nanoscale morphology and crystallinity. The DES-mediated approach provides predictable tuning of surface chemistry, leading to enhanced thermal stability and modulated interparticle interactions without compromising structural integrity.

A central outcome of this work is the identification of two concentration-dependent regimes that dictate CNC behavior and biointerface performance, revealing the continuum from colloidal dispersions to hydrogel networks within a single nanomaterial system. In the dilute colloidal regime, CNC–COOH forms stable dispersions with minimal aggregation, exhibiting negligible cytotoxicity toward 3T3-L1 fibroblasts and low-to-mild cytotoxic effects toward HT-29 epithelial cells. In contrast, at higher concentrations (2 wt %), CNC–COOH undergoes a transition into percolated hydrogel networks, particularly in ion-rich biological media as it is cell culture media, where ionic screening and hydrogen bonding promote the formation of soft, elastic structures capable of supporting 3D cell environments.

Importantly, these hydrogel networks sustain high cell viability (>74–98.4%) and enable 3D cellular organization, highlighting that cytocompatibility is not an intrinsic material property, but an emergent feature governed by concentration and material state. The absence of significant protein adsorption further indicates that network formation is primarily driven by physicochemical interactions rather than biomolecular corona effects.

Overall, this work establishes a direct link between sustainable surface modification, ionic content and concentration-dependent nanoscale assembly, and biological response. These insights provide a framework for the rational design of nanocellulose-based systems across colloidal and hydrogel states. While the enhanced cytocompatibility observed in CNC–COOH hydrogels is likely associated with their 3D architecture, surface carboxyl functionalities, and physicochemical microenvironment, elucidating the molecular and cellular mechanisms governing these responses remains an important direction for the future development of nanocellulose-based biointerfaces, 3D cell culture platforms, and biofabrication technologies.

## Supplementary Material



## Data Availability

Data will be made available on request.

## Notes

Conductometric titration, XPS deconvolution analyses, AFM characterization, TGA, DLS and zeta potential measurements, comparative dimensional analysis of CNCs, and additional photographs and spectra supporting the physicochemical characterization of CNC–COOH systems (PDF)
