# Supplementary material for: From Colloids to Hydrogels: Concentration-Dependent Cytocompatibility of Carboxylated Cellulose Nanocrystals Prepared via Deep Eutectic Solvents
Source: Langmuir. 2026 Jun 9;42(24):17658–71. doi: 10.1021/acs.langmuir.6c02021 (PMC13296517; doi:10.1021/acs.langmuir.6c02021)
Supplement: Supplementary file 1 [file la6c02021_si_001.pdf]

Supplementary Information

From Colloids to Hydrogels: Concentration-Dependent Cytocompatibility of Carboxylated Cellulose Nanocrystals Prepared via Deep Eutectic Solvents

Raúl Ortega-Córdova[a], Griselda Blanco-Gutiérrez[b], Priscila Quiñonez-Angulo[c,b], Francisco J. Flores-Ruiz[d], Kaori Sánchez-Carrillo[b], J. Félix Armando Soltero-Martínez[a], María G. Pérez-García[e], Karla Juarez-Moreno[b,] and Josué D. Mota-Morales[b,*]*

[a]Centro Universitario de Ciencias Exactas e Ingenierías, Universidad de Guadalajara, Guadalajara, Jalisco, 44430, México.

[b]Universidad Nacional Autónoma de México, Centro de Física Aplicada y Tecnología Avanzada, Querétaro, Querétaro, 76230, México.

[c]Department of Chemistry and Biochemistry, The Ohio State University, Columbus, Ohio 43210, USA.

[d]SECIHTI-Instituto de Física, Benemérita Universidad Autónoma de Puebla, Ciudad Universitaria, Puebla 72570, México.

[e]Centro Universitario de Tonalá, Universidad de Guadalajara, Tonalá, Jalisco, 45425, México.

[*]Corresponding authors: kjuarez@fata.unam.mx ; jmota@fata.unam.mx

Supporting Information includes conductimetric titration, XPS deconvolution analyses, AFM characterization, TGA, DLS and ζ-potential measurements,

comparative dimensional analysis of CNCs, and additional photographs and spectra supporting the physicochemical characterization of CNC–COOH systems.

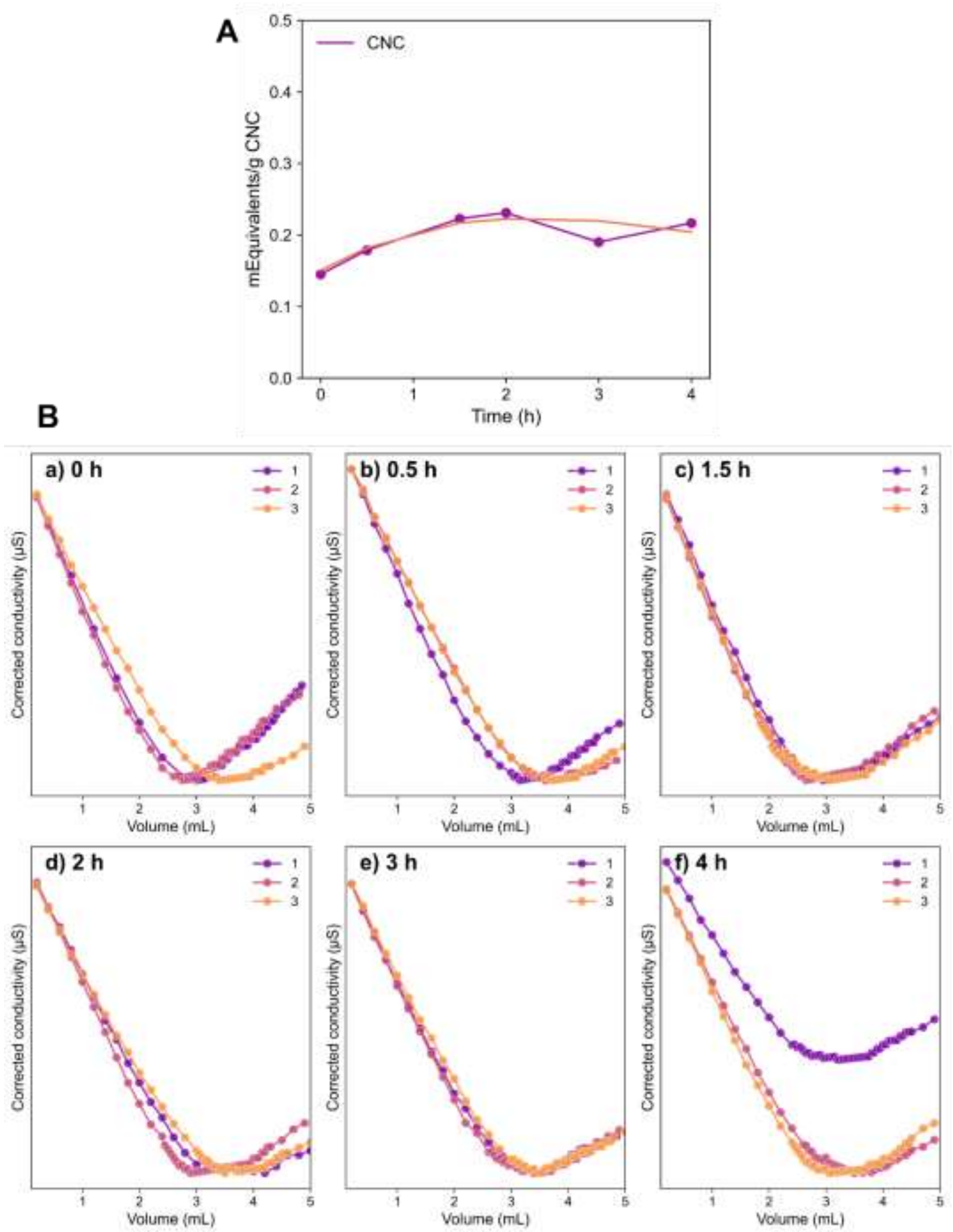

Figure S1. A) Milliequivalents per gram of functionalized CNC as a function of reaction time. B) Conductometric titration curves obtained in triplicate for each reaction time.

Table S1. Milliequivalents per gram of functionalized CNCs as a function of reaction time.

Reaction times	mEquivalents gCNC^{-1}
0	0.145 ± 0.035
0.5	0.179 ± 0.025
1.5	0.223 ± 0.045
2	0.231 ± 0.042
3	0.190 ± 0.012
4	0.217 ± 0.014

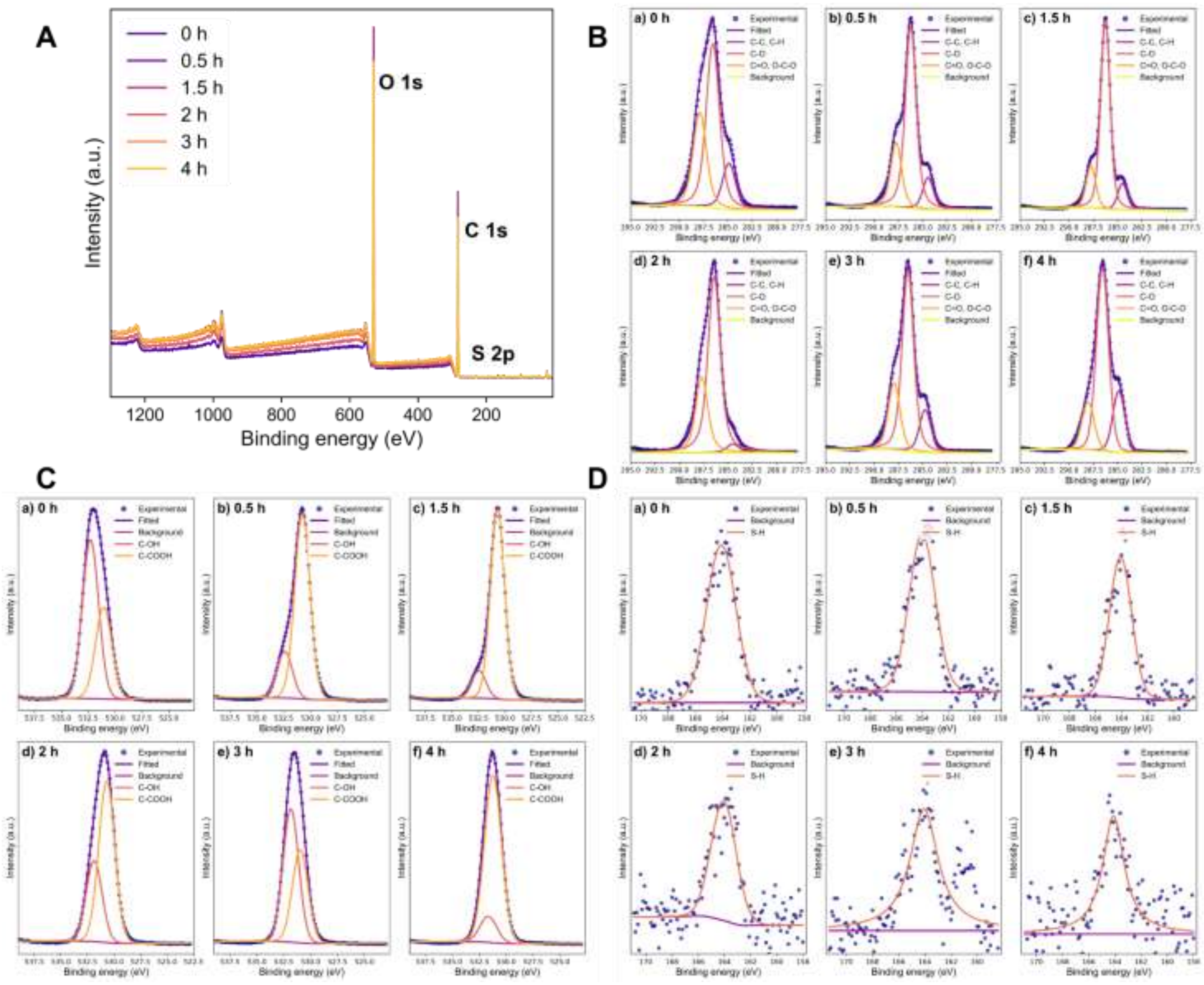

Figure S1. A) High-resolution XPS spectra for CNC samples at different reaction times with deconvoluted spectra for B) C *1s*, C) O *1s* and D) S *2p*.

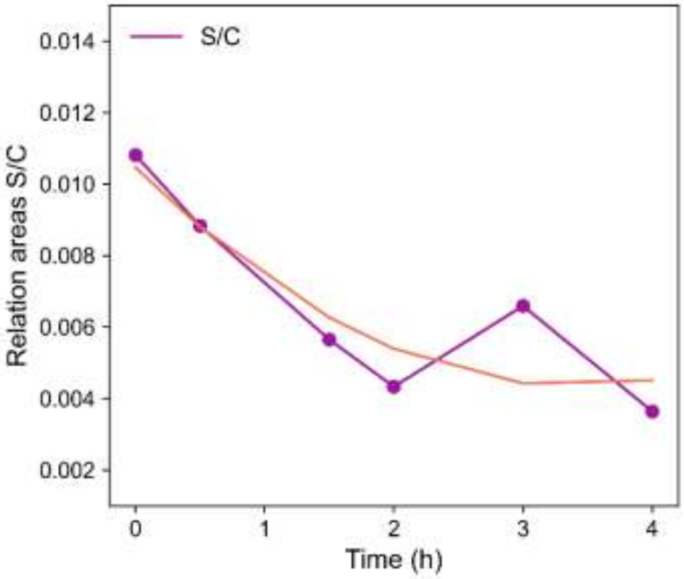

Figure S2. Sulfur-to-carbon (S/C) peak area ratio obtained from XPS analysis as a function of reaction time for functionalized CNCs.

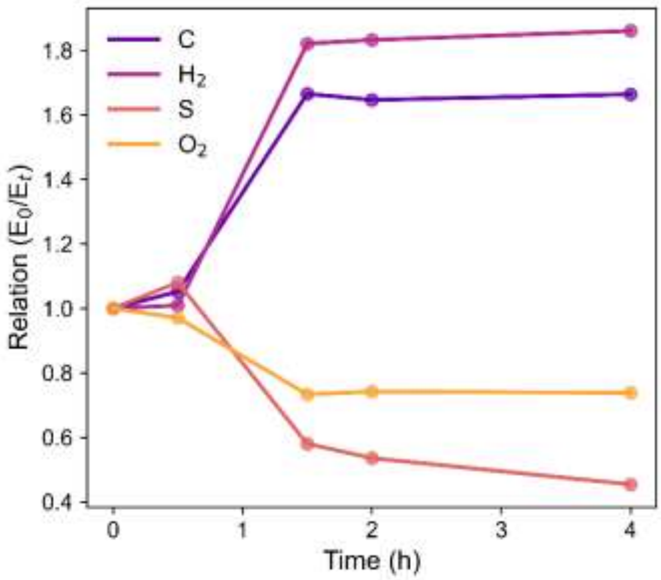

Figure S4. Relative ratio of elements in CNCs determined by XPS at 0 h and as a function of reaction time.

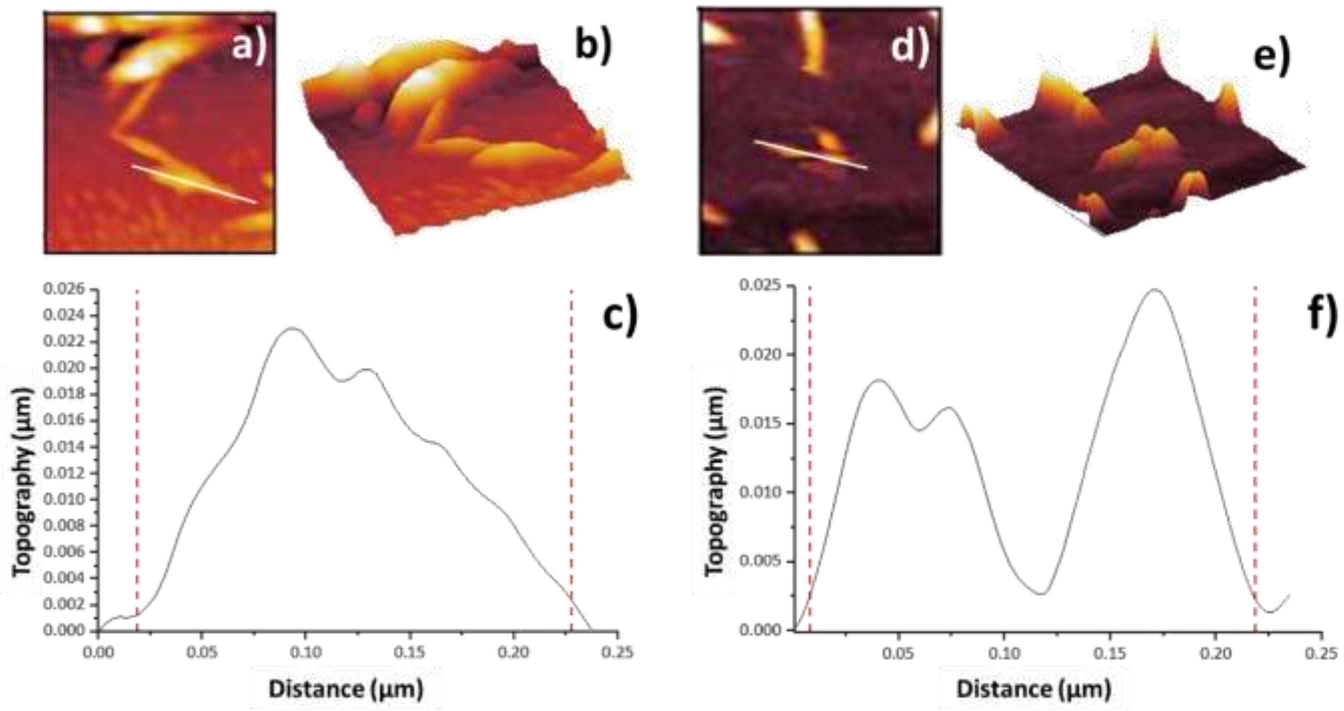

Figure S5. a) AFM image of a cellulose nanocrystal (0 h) in two dimensions with a length of 208 nm. b) Three-dimensional image of the CNC shown in image a). c) Height profile of the line drawn in a) and its length. d) AFM image of a cellulose nanocrystal (3 h) in two dimensions with a length of 207 nm. e) Three-dimensional image of the CNC shown in image d). f) Height profile of the line drawn in d) and its length.

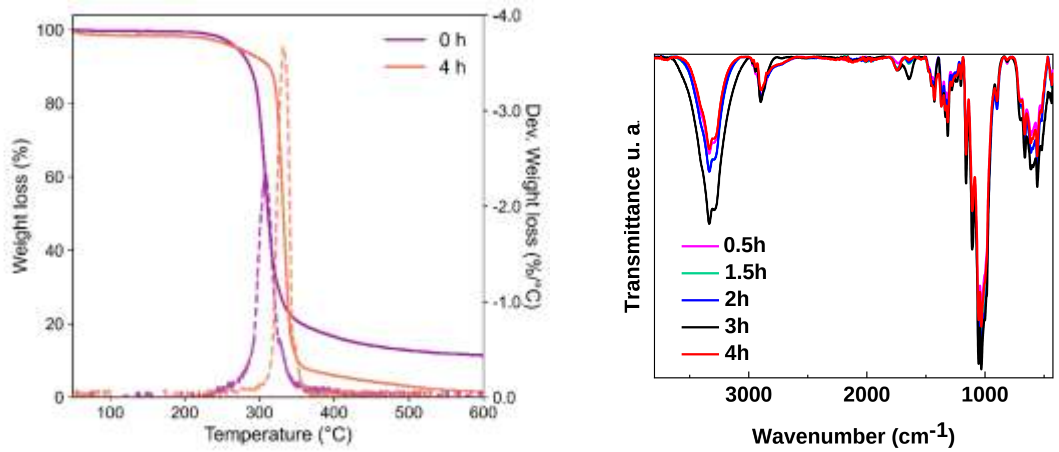

Figure S6. (Left) TGA thermograms of CNC-S (0 h) and CNC functionalized for 4 h. (Right) FTIR spectra of CNC functionalized at different times (0.5-4 h).

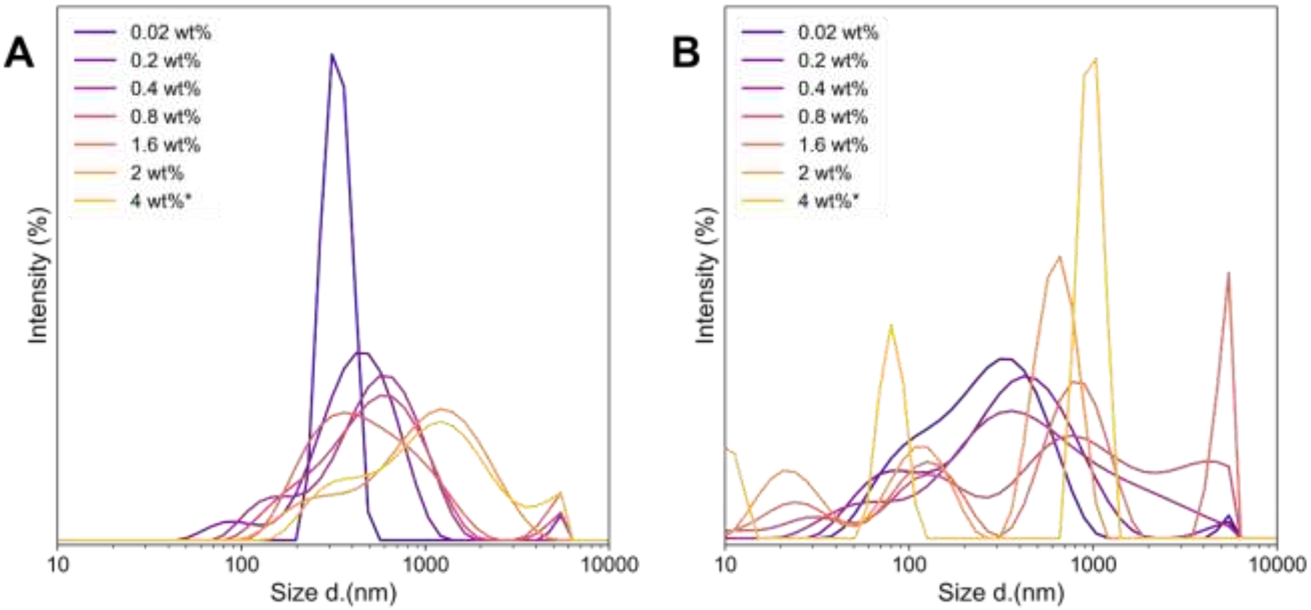

Figure S7. DLS measurements of A) CNC-S and B) CNC-COOH at different weight percent concentrations in water.

Table S2. Dimensions comparison of CNCs

Technique	CNC-S (nm)	CNC-COOH (nm)	Reference
AFM	208<br>379.4<br>-	207<br>288.3<br>330 ± 106	This work<br>Reference [1]<br>Reference [2]
DLS	195 ± 30.3	323 ± 38.6	This work
TEM	-<br>-<br>-<br>-	337 ± 23<br>400 - 600<br>180 ± 20<br>150 - 175	Reference [3]<br>Reference [4]<br>Reference [5]<br>Reference [6]

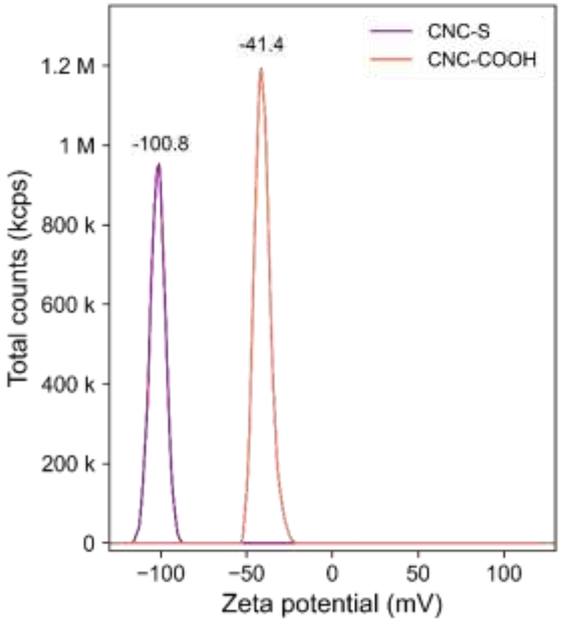

Figure S8. Zeta potential measurement for sulfated (CNC-S) and carboxylated (CNC-COOH) cellulose nanocrystals. The presented result is an average of three independent measurements.

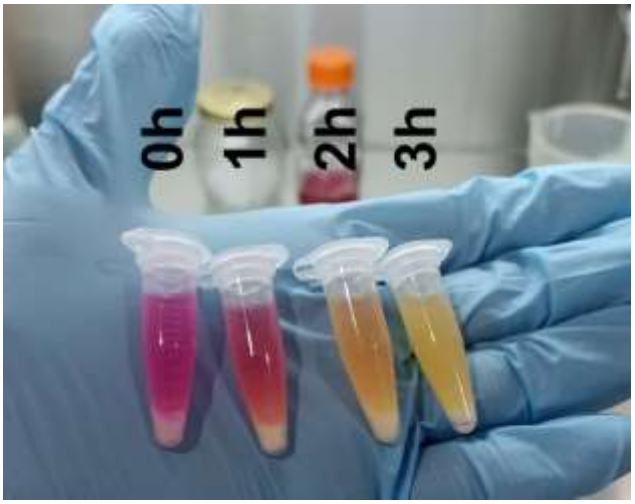

Figure S9. Coloration of CNC samples in DMEM before washing with PBS.

References

(1) Wei, Z.; Wang, D.; Chen, Y.; Yu, D.; Ding, Q.; Li, R.; Wu, C.; Wei, Z.; Wang, D.; Chen, Y.; Yu, D.; Ding, Q.; Li, R.; Wu, C. The H-Bond Evolution of Cellulose Nanofibrils Treated with Choline Chloride/Oxalic Acid. *Cellulose 2022 29:7* **2022**, *29* (7), 3675–3687. https://doi.org/10.1007/S10570-022-04517-6.

(2) Jiang, J.; Li, Z.; Luo, J.; Meng, J.; Cheng, L.; Qin, H. The Morphological Regulation of Nanocellulose Using a Hydrated Deep Eutectic Solvent System. *Cellulose* **2025**, *32* (12), 7145–7157. https://doi.org/10.1007/s10570-025-06667-9

(3) Sirviö, J. A.; Visanko, M.; Liimatainen, H. Acidic Deep Eutectic Solvents As Hydrolytic Media for Cellulose Nanocrystal Production. *Biomacromolecules* **2016**, *17* (9), 3025–3032. https://doi.org/10.1021/acs.biomac.6b00910

(4) Wang, H.; Li, J.; Zeng, X.; Tang, X.; Sun, Y.; Lei, T.; Lin, L. Extraction of Cellulose Nanocrystals Using a Recyclable Deep Eutectic Solvent. *Cellulose* **2020**, *27* (3), 1301–1314. https://doi.org/10.1007/s10570-019-02867-2

(5) Zhou, J.; Abdalkarim, S. Y. H.; Chen, X.; Yu, H-Y. Sustainable and General Production of Carboxylated Cellulose Nanocrystals via Synergistic Lewis-Acid/Organic-Acid Hydrolysis under Ambient Conditions. *ACS Sustainable Chem. Eng.* **2025**, *13*, 16192−16203. https://doi.org/10.1021/acssuschemeng.5c08214

(6) Chen, Y.; Jia, B.; Zhou, J.; Jin, G.; Yu, H-Y. Dual pH/temperature-responsive cellulose nanocrystals with tailored dual-triggered structural adaptability for controlled-release oral tablet. *Int. J. Biol. Macromol.* **2026**, 350, 150979. https://doi.org/10.1016/j.ijbiomac.2026.150979
